# Accelerated large-scale multiple sequence alignment

**DOI:** 10.1186/1471-2105-12-466

**Published:** 2011-12-07

**Authors:** Scott Lloyd, Quinn O Snell

**Affiliations:** 1 Computer Science Department, Brigham Young University, Provo, UT 84602, USA

## Abstract

**Background:**

Multiple sequence alignment (MSA) is a fundamental analysis method used in bioinformatics and many comparative genomic applications. Prior MSA acceleration attempts with reconfigurable computing have only addressed the first stage of progressive alignment and consequently exhibit performance limitations according to Amdahl's Law. This work is the first known to accelerate the third stage of progressive alignment on reconfigurable hardware.

**Results:**

We reduce subgroups of aligned sequences into discrete profiles before they are pairwise aligned on the accelerator. Using an FPGA accelerator, an overall speedup of up to 150 has been demonstrated on a large data set when compared to a 2.4 GHz Core2 processor.

**Conclusions:**

Our parallel algorithm and architecture accelerates large-scale MSA with reconfigurable computing and allows researchers to solve the larger problems that confront biologists today. Program source is available from http://dna.cs.byu.edu/msa/.

## Background

Biologists and other researchers use multiple sequence alignment (MSA) as a fundamental analysis method to find similarities among nucleotide (DNA/RNA) or amino acid (protein) sequences. The compute time for an optimal MSA grows exponentially with respect to the number of sequences. Consequently, producing timely results on large problems requires more efficient algorithms and the use of parallel computing resources. Reconfigurable computing hardware, such as Field-Programmable Gate Arrays (FPGAs), provides one approach to the acceleration of biological sequence alignment. Other acceleration methods typically encounter scaling problems that arise from the overhead of inter-process communication and from the lack of parallelism. Reconfigurable computing allows a greater scale of parallelism using many fine-grained custom processing elements that have a low-overhead interconnect.

The most common algorithm used to solve the MSA problem is progressive alignment [1-3]. This algorithm consists of three main stages. The first stage compares all the sequences with each other producing similarity scores only. Since this stage is easily parallelized, it has traditionally been the focus of parallelization efforts; however, speedup is limited without accelerating the following stages. The second stage of MSA groups the most similar sequences together using the similarity scores to form a tree that guides alignment in the next stage. Finally, the third stage successively aligns the most similar sequences and groups of sequences until all the sequences are aligned. Groups of aligned sequences are converted into profiles before alignment with a pairwise dynamic programming algorithm. A profile represents the character frequencies for each column in an alignment. In Stage 3, traceback information from full pairwise alignment is required to align groups of sequences.

Accelerator technology requires moving data from the host address space to the accelerator before computation. If the computation rate on the accelerator exceeds the communication rate with the host, performance will be limited. Ideally, the communication rate is at least equal to or greater than the computation rate. FPGAs are capable of handling parallel computations on many small integer data types; however, floating-point operations require more resources and consequently fewer operations fit within the same logic. Reducing complex profiles to a simpler integer form allows greater performance on the accelerator by lowering the needed communication rate and permitting more processing elements.

In this work, a new method for accelerating the third stage is described that reduces subgroups of aligned sequences into discrete profiles before they are pairwise aligned on the accelerator. Our pairwise alignment algorithm [4] produces the required traceback information and does not limit the sequence length by the number of processing elements (PEs) or by the amount of block RAM on the accelerator. Other hardware acceleration methods are inadequate for use in the third stage because the sequence length is severely limited or only similarity scores are computed.

Alignment quality of the new method is assessed with the BRAliBase benchmark RNA alignment database [5] that consists of 18,990 RNA alignments and with the MDSA data set [6]. Discrete profile alignment is shown to have comparable quality to other popular MSA programs and an accelerated version of the program demonstrates two orders of magnitude speedup.

### Related Work

Most efforts to accelerate bio-sequence applications with hardware have focused solely on database searches and have employed a pairwise local comparison algorithm. Ramdas and Egan [7] discuss several FPGA-based architectures in their survey. Other pairwise comparison accelerators have also been described in [8-10]. A few methods to accelerate MSA with hardware have been demonstrated, but they fail to use all the available parallel resources in every stage of MSA; consequently, performance is reduced in some stages with idle processors.

Without accelerating the most time consuming stages of progressive MSA, Amdahl's law [11] limits the overall speedup. For example, if the third stage takes 5% of the computation time, the overall speedup is limited to about 20 even if the other stages are infinitely fast. If the time in Stage 1 is reduced with faster comparison techniques, then the acceleration of Stage 3 becomes more critical. Newer programs like MUSCLE [12] and MAFFT [13] use a faster alignment-free comparison method; therefore, the third stage usually dominates the computation time. Even though these newer methods show greater performance, most of the related work has still focused on accelerating ClustalW where the first stage dominates the run time.

Multiprocessor-Supercomputer

Most attempts to accelerate MSA have been on shared-memory or distributed-memory systems using a coarse-grained parallel approach. Mikhailov et al. [14] shows a 10x speedup with 16 processors by parallelizing all three stages of ClustalW [3] with OpenMP [15] on a shared-memory SGI Origin machine. A notable feature of this effort is the parallelization of the guide tree calculation in the second stage. Deng et al. [16] parallelized several stages of MUSCLE [12] to realize a speedup of 15 on a 16 processor shared-memory machine. Several attempts [17-20] have been made to parallelize ClustalW on distributed-memory systems using message passing. In these cases, Stages 1 and 3 were parallelized with the best performance reported by Lin et al. [21] having a speedup of 29 on 64 CPUs. Tan et al. [22] achieved a speedup of 35 on a hybrid multiprocessor-cluster system of 40 nodes with 80 CPUs. In the third stage, Tan's method distributes group-to-group alignments to system nodes using a method that is based upon guide-tree and recursive parallelism. The main contribution comes from computing the forward and backward DP scans in parallel on processors within a node. The small speedup achieved in the third stage, which is under 10 in most cases, limits the overall speedup of the progressive algorithms.

Cell BE

Recently, the Cell Broadband Engine has received attention as an accelerator for MSA. Vandierendonck et al. [23] have accelerated ClustalW by a factor of 8 when compared with a 2.13 GHz Intel Core2 Duo processor running a single thread. Stages 1 and 3 were parallelized on two Cell BEs by vectorizing DP matrix calculations and scheduling independent tasks across the 16 available synergistic processing elements. Using a Playstation3, Wirawan et al. [24] achieved a peak speedup of 108 on the first stage when compared with a 3.0 GHz Pentium 4. Overall, a speedup of only 13.7 was observed on 1000 sequences with an average length of 446. However, the announcement from IBM to discontinue Cell production for technical computing [25] may diminish further interest in the Cell.

GPU

Another popular acceleration technology is the general purpose graphics processing unit. Its commodity nature has sparked much interest outside of the graphics community as an acceleration engine. Liu et al. [26] accelerated all three stages of ClustalW on the GPU, with the parallel portions programmed using CUDA [27]. When independent task and guide tree parallelism is low, cells of DP matrix calculations are computed in parallel. An overall peak speedup of 41.53 was demonstrated on 1000 sequences of average length 858 with 1 GPU card (GeForce GTX 280) when compared with a 3.0 GHz Pentium 4. The best speedup obtained in each of the three stages is 47.13, 11.08, and 5.9 respectively. Again, the small gain in the third stage limits the overall speedup.

FPGA

Reconfigurable computing approaches accelerate the first stage of MSA by computing pairwise alignments with a pipeline of processing elements (PEs). This linear systolic array operates with fine-grained parallelism along a wavefront of cells in the DP matrix. The ClustalW algorithm does not use the score obtained from a pairwise alignment directly. Instead, the number of identical characters in an alignment are used to compute the fractional identity. Oliver et al. [28] accelerates the first stage of ClustalW, but leaves the second and third stages for execution on the host processor. Instead of actually aligning the sequences, a custom algorithm on the accelerator counts the number of identical characters during the forward scan without performing traceback. The best overall speedup was 13.3 compared to ClustalW running on a 3.0 GHz Pentium 4. For Stage 1, a PCI-based accelerator board reaches a peak speedup of 50.9 with 92 PEs in a Xilinx XC2V6000. In another approach, Lin et al. [21] demonstrated an overall speedup of 34.6 using 10 Altera Stratix PEIS30 with a total of 3072 PEs. For the first stage, a speedup of 1697.5 was achieved when compared with a 2.8 GHz Xeon. The number of identical characters is deduced from the comparison score returned from the accelerator and the sequence lengths. Even with the impressive speedup in the first stage, the overall speedup is still limited by the third stage. Greater performance may be achieved, however, by accelerating the third stage of progressive alignment.

## Methods

### Discrete Profile Alignment

**Figure 1:**
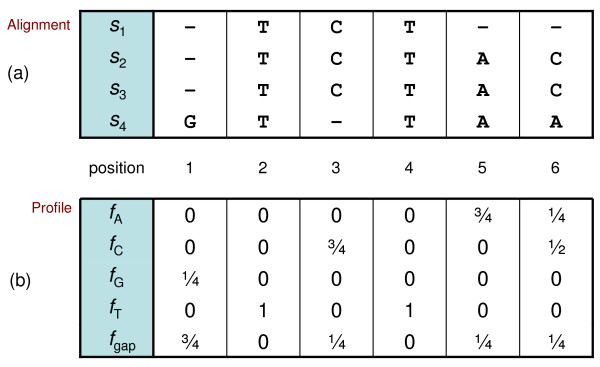
**Example multiple alignment and derived profile**. Each position in a profile consists of a vector with character frequencies *f_N _*for the corresponding column in a group of aligned sequences. (a) Multiple alignment of sequences *s_i_*. (b) Profile derived from the alignment.

**Figure 2:**
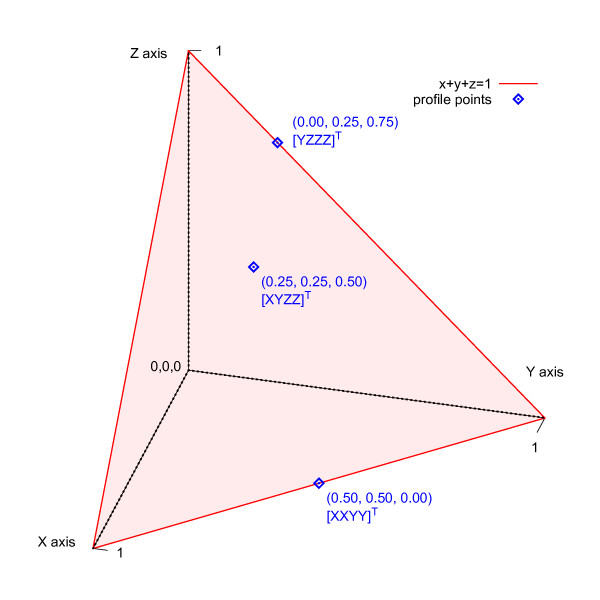
**Profile space**. In three dimensions, profile space is a triangle on the plane *x *+ *y *+ *z *= 1; however, five dimensions are required to represent DNA alignments. Points in profile space are shown with coordinates and an aligned column example (transposed). The corners of profile space represent columns of an alignment that contain all the same character.

The third stage of MSA pairwise aligns profiles in a similar way to sequences, but it must also work with the extra information in profiles. Each position of a profile designates a point in continuous profile space with a vector of character frequencies (see Figure 1 and 2). Profile-based MSA applications typically use floating-point numbers or scaled integers to represent these character frequencies. The extra size and dimension of profiles, in relation to sequences, adds to the complexity of alignment. Hence, a reduced representation of profiles that retains as much information as possible simplifies alignment. By reducing profiles to discrete profiles--essentially sequences with an extended alphabet--they may be aligned with a simpler, higher-performing, pairwise sequence alignment algorithm.

The concept of discrete profile space was introduced by Eskin [29] with application to DNA motif search, which finds relatively short patterns in a subject sequence. For instance, when searching for promoter sequences, query profiles have a length of about 8-12 positions. In Eskin's method, a motif is represented as a small, discrete profile that contains the probabilities of finding each nucleotide at the respective positions. A similar work by Wang and Stormo [30] partitions a four-dimensional continuous profile space into 15 subspaces based upon a supervised learning algorithm. Each dimension corresponds to a nucleotide frequency *f_N _*_= {A, C, G, T} _with the constraint Σ *f_N _*= 1. Any point falling within a partition is then represented by a discrete profile symbol.

For the application of discrete profile space to MSA, a few issues and extensions must be addressed. For example, an additional dimension must be added to profile space to accommodate gaps. Also, sample points from profile space must be selected for representation with discrete symbols, and substitution costs need to be calculated between these sample points. Furthermore, a reduction method from continuous space to discrete symbols must be devised that can operate efficiently on genomic-sized profiles.

#### Sample Points

Five dimensions in profile space are required to represent profiles that contain nucleotide and gap character frequencies. Each position of a profile can be mapped to a point that falls on the bounded hyperplane *f_A _*+ *f_C _*+ *f_G _*+ *f_T _*+ *f_gap _*= 1 in Euclidean space where 0 ≤ *f_N _*≤ 1 and *N *= {A, C, G, T, "-"}. To reduce the number of possible points, a discrete number of sample points are selected from continuous profile space. These sample points and a corresponding discrete symbol represent nearby points in profile space.

**Figure 3:**
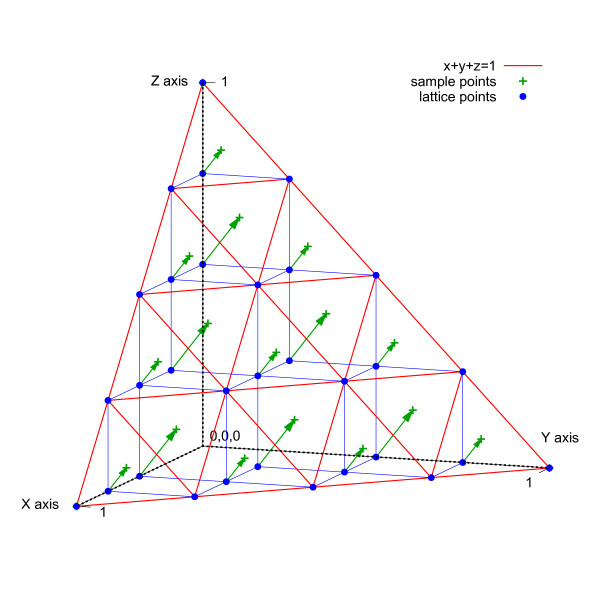
**Sample point determination**. Sample points are determined by projecting lattice points onto the profile plane.

**Figure 4:**
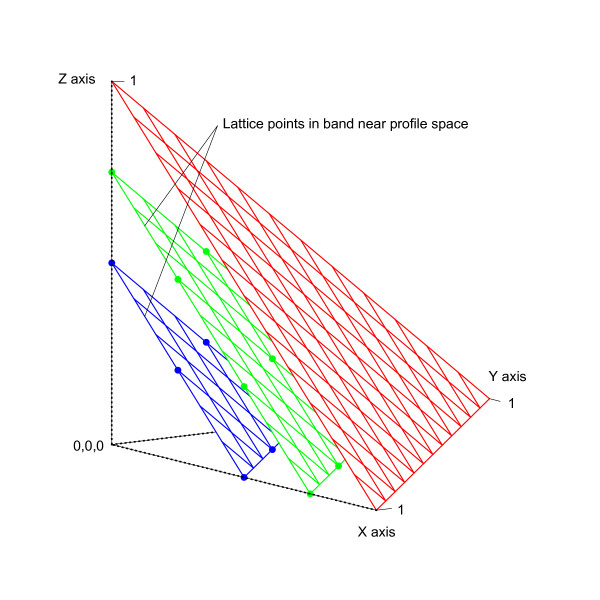
**Planes parallel to profile space**. Planes parallel to profile space are separated by a distance of $\varepsilon =\sqrt{D}∕DL$. For this example, *D *= 3 and *L *= 4.

Given *D *dimensions, a selection algorithm determines sample points by projecting lattice points ***p ***in *D*-space onto the profile hyperplane according to the parametric equation ***p'***= *t**p***, where *t *= (1 *- *Σ*p_i_*)*/D*. Let *L *denote the number of lattice points on the interval [0,1] of each axis. Lattice points (see Figure 3) are evenly spaced by a distance of 1*/L *in each dimension; however, only points that lie in a band near the hyperplane are considered. Given the sum of lattice point coordinates *S *= Σ*p_i_*, the considered points fall between $\left(1-\frac{D-1}{L}\right)\leq S\leq \left(1-\frac{1}{L}\right)$. Intuitively, these lattice points reside on parallel hyperplanes that are a distance of $\varepsilon =\sqrt{D}∕DL$ from each other (see Figure 4). Corners of profile space that consist of all one nucleotide are also included as sample points, but the point indicating a profile of all gaps is excluded.

The number of sample points is reduced further by filtering points that represent less probable nucleotide frequencies. Nucleotides from the same group, either purine or pyrimidine, have a higher probability of being aligned, while those from different groups have a lower probability. Substitution tables reflect this probability in their cost values and influence alignment algorithms accordingly. Therefore, sample points with a high frequency of both purines and pyrimidines are eliminated if they meet the condition

$$\left(f_{i}+f_{j}>T_{c}\right)\wedge \left(|f_{i}-f_{j}|\;<T_{d}\right)$$

where *i *∈ {A, G} and *j *∈ {C, T}. The first term asserts that the combined frequency of purines and pyrimidines is above a threshold *T_c_*, and the second term asserts that both groups have similar frequencies with a difference less than *T_d_*. Threshold values can be adjusted to allow more or less sample points depending on the desired number of discrete symbols. Effective starting values for the thresholds are *T_c _*= 0.75 and *T_d _*= 0.30.

#### Substitution Table

After sample points in profile space are selected, the substitution cost between these representative points can be determined. Instead of calculating the cost every time sample points are compared during alignment, the cost can be computed once and stored in a new sample substitution table. The discrete symbols associated with each sample point become the indices into the table and the codebook for a quantization algorithm.

Substitution costs between sample points are computed from the individual nucleotide frequencies and substitution costs. Since a hardware constrained implementation of the sample substitution table may only have 4 or 8-bit entries, a scaling factor adapts the range of computed values to fit within entry size limits. Given the nucleotide substitution table *s *of size *N *× *N*, an array of sample points *c*, and a scaling factor *β*, the substitution cost *s' *between each sample point is determined by

$$s_{i,j}^{\prime }=\left[\overset{N}{\underset{m\;=\;1}{\sum }}\overset{N}{\underset{n\;=\;1}{\sum }}c_{j,\;n}\;c_{i,\;m}\;s_{n,\;m}\right]\;\beta .$$

The substitution cost of a gap and a nucleotide is the gap extension cost plus one. This prevents a gap in one sequence from being followed by a gap in the other sequence during pairwise alignment of discrete profiles.

#### Reduction

**Figure 5:**
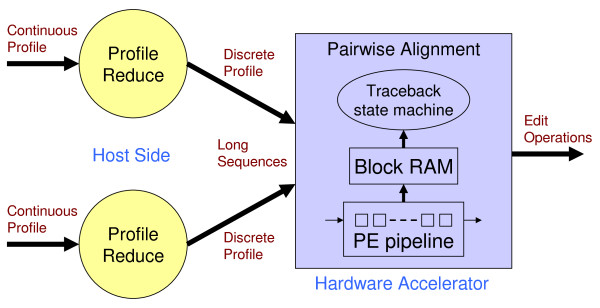
**Profile reduction before alignment**.

For the accelerator to sustain maximum performance, the host system must supply reduced profiles at the accelerator's input data rate (see Figure 5). Profiles are reduced to discrete profiles to support a simpler, higher-performing pairwise alignment algorithm on an accelerator that only aligns sequences of symbols. A new quantization technique is used for this reduction on the host to reach the needed performance. For each continuous profile position, the reduction algorithm searches for a nearby sample point and then returns the corresponding discrete symbol. Finding a nearby point in less time is preferred to a nearest neighbor search with greater overhead. Also, constraining the search to the profile hyperplane Σ *f_N _*= 1 allows for some optimization.

A near neighbor search finds a sample point that is close to the given continuous point, but not necessarily the closest point. This relaxation of proximity allows the search to proceed in deterministic time, and thereby keep up with the accelerated pairwise alignment. Search begins by scaling and truncating each nucleotide frequency to form a partially quantized point. Then these integral coordinates are used as indices into a lookup table *R *that contains references to nearby sample points. The scale factor determines the number of quantization levels for each coordinate and also the size of the lookup table. As a result of the search, points in continuous profile space are mapped to a small set of symbols that represent sample points. Not every element of the *D*-dimensional lookup table requires storage since the partially quantized points lie within a scaled distance of (*D *- 1)*ε *from the profile hyperplane. A ragged array with only the needed locations is used to implement the lookup table *R*.

#### Example

An example of discrete profile alignment is presented starting with two groups of aligned sequences. Profile formation, reduction, and pairwise alignment of the profiles are included in this example. A simplified alphabet Σ*' *= {A, C, "-"} is used so that the character frequencies correspond with the X, Y and Z axes of a depictable three-dimensional profile space.

**Figure 6:**
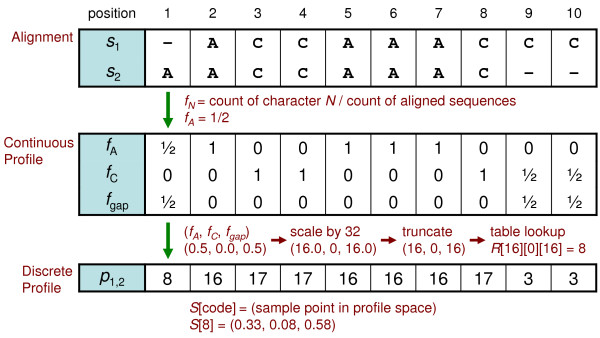
**Example profile calculation and reduction for sequences 1 and 2**. From the alignment {*s*_1_, *s*_2_}, a continuous profile is derived and then reduced to form the corresponding discrete profile *p*_1,2_. *S *is a table of sample points.

**Figure 7:**
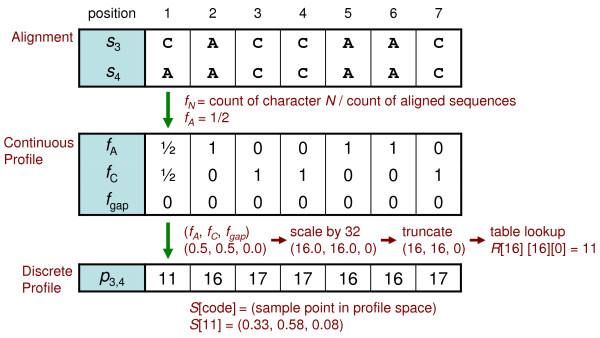
**Example profile calculation and reduction for sequences 3 and 4**. From the alignment {*s*_3_, *s*_4_}, a continuous profile is derived and then reduced to form the corresponding discrete profile *p*_3,4_. *S *is a table of sample points.

**Figure 8:**
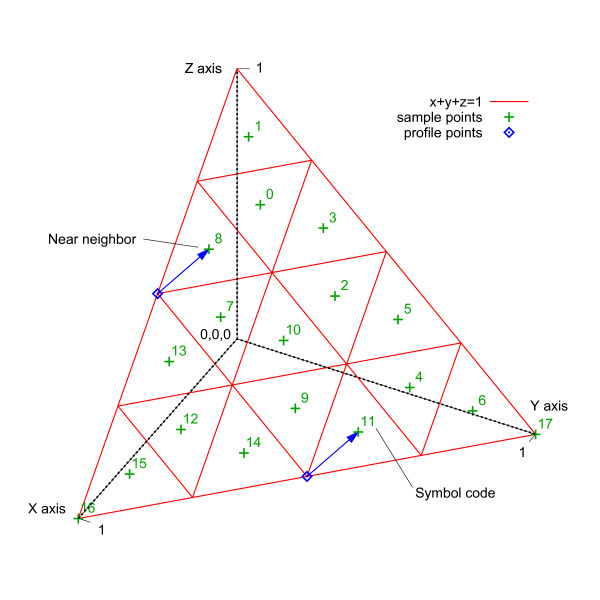
**Near neighbors in profile space**. Given two profile points, nearby sample points and associated symbol codes are shown.

**Figure 9:**
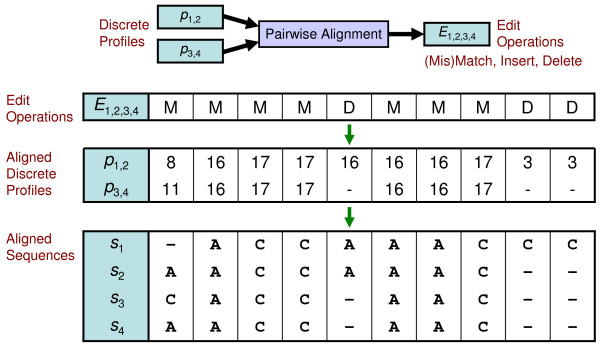
**Example profile alignment**. A pairwise alignment algorithm treats discrete profiles as sequences. The resulting edit operations *E*_1,2,3,4 _indicate the computed alignment between the discrete profiles *p*_1,2 _and *p*_3,4_, and the corresponding groups of sequences {*s*_1_, *s*_2_} and {*s*_3_, *s*_4_}.

Figures 6 and 7 show instances of profile calculation and reduction. Each profile position is calculated independently and corresponds with a column of aligned sequences. Given two groups of sequences {*s*_1_, *s*_2_} and {*s*_3_, *s*_4_}, continuous profiles are calculated by counting the occurrence of characters in each column and dividing by the number of sequences to produce a vector of frequencies (*f_A_*, *f_C_*, *f_gap_*). Profile reduction proceeds by scaling each vector and truncating the values to form indices into the three-dimensional reduction table *R_A_*_,*C*,*gap*_. In this example, the scale factor is equivalent to the size of *R *in each dimension. Table lookup values, which are references to nearby sample points, are used for each position of the discrete profiles *p*_1,2 _and *p*_3,4_. Figure 8 depicts two points in profile space and the nearby sample points found by lookup in the reduction table *R*. Less probable sample points are not removed in this instance. Figure 9 shows the discrete profile alignment process and the final alignment of the original groups. The discrete profiles *p*_1,2 _and *p*_3,4 _are aligned with a pairwise algorithm that returns the edit string *E*_1,2,3,4 _composed of the operations *e_i _*∈ {(Mis)Match, Insert, Delete}. This edit string also applies to the groups of sequences {*s*_1_, *s*_2_} and {*s*_3_, *s*_4_} because of the position correspondence between alignments and derived profiles.

### Experimental Setup

The following components were incorporated into MUSCLE [12], an open-source MSA program, to demonstrate accelerated large-scale MSA.

• SSE accelerated sequence similarity algorithms for the first stage of MSA

• A discrete profile alignment algorithm for the third stage of MSA

• An FPGA accelerated pairwise alignment algorithm [4]

Corresponding code in MUSCLE was replaced with our highly-parallel code that uses SSE instructions and the FPGA accelerator. Discrete profile alignment replaced the float-based alignment used in each step of progressive alignment. Whenever two continuous profiles are aligned, discrete profiles are first computed and then aligned.

Pairwise sequence alignment is a fundamental subcomponent of the discrete profile alignment algorithm. Sequences are aligned on the FPGA accelerator with a space-efficient dynamic programming algorithm and a traceback procedure. Given two sequences, the accelerator returns an edit string that describes an optimal alignment. This alignment functionality is incorporated into a C library that is called from the host platform.

MUSCLE uses different methods to calculate sequence similarity in the first and second iterations. Both of these methods have been optimized with SSE instructions. The first method does not compare sequences directly, but instead compares two vectors that contain k-mer counts from corresponding sequences. These vectors are compared 16 elements at a time with SSE instructions to find the minimum values. The second method determines the percent identity of two aligned sequences by counting the number of matched symbols. To accelerate this method, SSE instructions compare 16 symbols from each sequence at once and increment corresponding counts in a vector register.

Two versions of the modified MUSCLE are used for analysis. One version (MUDISC) implements our pairwise alignment in software on the host, while the other (MUFPGA) accelerates pairwise alignment on the FPGA. MUDISC is compared with other popular MSA programs such as ClustalW [3], Kalign [31], MAFFT [13], MUSCLE [12], and POA [32]. Program version numbers and command-line options are shown in Table 1. For those programs that support iterations, a maximum of two are used. The non-accelerated MSA programs and MUDISC execute only on the conventional processor and MUFPGA additionally uses the FPGA accelerator. Both alignment quality and program performance are measured.

**Table 1:** Program version numbers and command-line options

Version	Program	Options
1.83	ClustalW	-type = dna
2.04	Kalign	--nuc
6.811	MAFFT	--nuc
3.8.31a	MUDISC	-seqtype rna -maxiters 2
3.8.31a	MUSCLE	-seqtype rna -maxiters 2
2	POA	-do_global -do_progressive

MUDISC and MUSCLE additionally use the option "-termgaps full" on BRAliBase and "-termgaps ext" on MDSA when assessing alignment quality. MUDISC, MUFPGA, and MUSCLE use the options "-termgaps full -seqtype dna -gapopen 0 -center -300 -maxiters 2" for the performance test.

Nucleotide adaptations [6] of the BAliBASE [33] and SMART [34] reference alignments are used to compare the quality of the MSA programs. BAliBASE alignments have been determined to be correct based upon known three-dimensional structure. Another assessment of alignment quality is obtained with the BRAliBase benchmark RNA alignment database [5] that consists of 18,990 RNA alignments. Unaligned versions of the reference alignments are realigned to produce test alignments. Reference and test alignments are then compared with the scoring programs MSCORE and SCIF to produce an accuracy metric between 0 and 1. The MSCORE program combines several scoring methods, which are described in the Results section.

A performance analysis uses a few large-scale, viral data sets that range in average length up to 167,043 nucleotides or contain up to 12,104 sequences. Overall program performance for MUSCLE, MUDISC, and MUFPGA is measured by the wall-clock time needed to align a data set and includes all three stages of progressive alignment. For accurate timing, the host processor's performance counters are used.

The host platform consists of a desktop computer with 8 GB of DRAM and a 2.4 GHz Intel Core2 Duo processor running 64-bit Fedora 13 Linux as the operating system. All benchmark applications execute in a single thread and are compiled with gcc using -O3 optimization. An 8-lane PCI Express [35] add-in card with a Xilinx Virtex-4 FX100 FPGA provides the hardware acceleration for pairwise alignment. Acceleration occurs on a pipeline of 256 PEs driven by a 100 MHz clock. Each PE requires one block RAM to implement the substitution cost $s_{i,j}^{\prime }$ as a lookup table. The accelerator supports linear gap costs and up to 64 points in profile space with 6-bit symbol values.

## Results and Discussion

**Figure 10:**
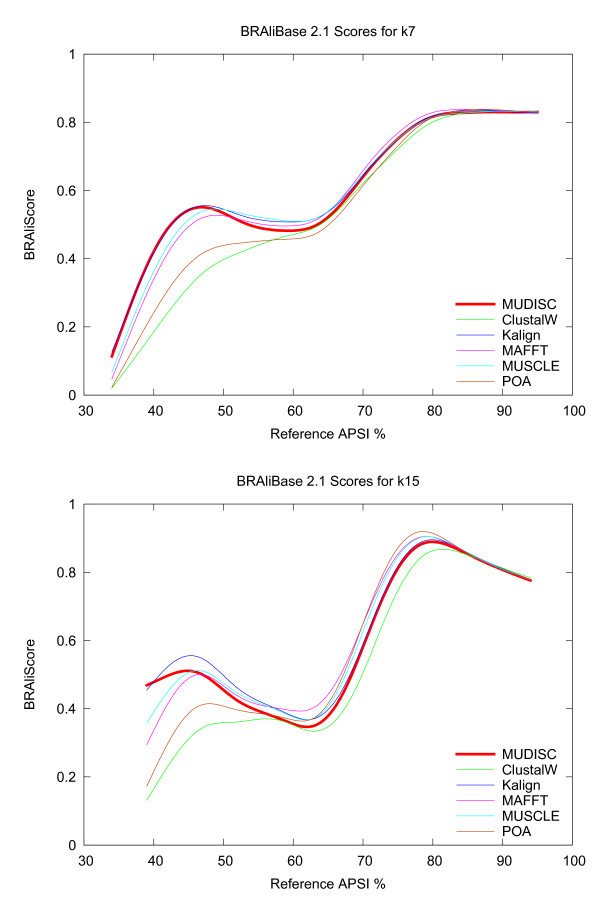
**Alignment quality on the BRAliBase data set**. MUDISC (the new method) is compared with several alignment programs on a seven (k7) and fifteen (k15) sequence RNA reference set from BRAliBase 2.1. A higher score indicates better quality and is shown in relation to the average pairwise sequence identity (APSI). MUDISC uses discrete profile alignment.

Alignment quality with BRAliBase 2.1 is depicted in Figure 10 for MUDISC and several other MSA programs. The BRAliScore, which reflects the alignment accuracy, is plotted in relation to the average pairwise sequence identity (APSI) of the reference alignment. Identical sequences have an APSI of 100%. BRAliScore is composed of two independent scores and is calculated by multiplying the fractional identity (FI) [36] and the structure conservation index (SCI) [37]. The FI score is based on the fraction of matching characters between the test and reference alignment, whereas the SCI is not based on the reference alignment, but indicates the amount of secondary structure conserved in the multiple alignment. A local smoothing of score values is applied with the acsplines option in gnuplot and a weighting factor of 5e-3. Above 60% APSI, there is little difference in the alignment quality between the programs; however, MUDISC is one of the top performers on data sets below 60% APSI.

**Figure 11:**
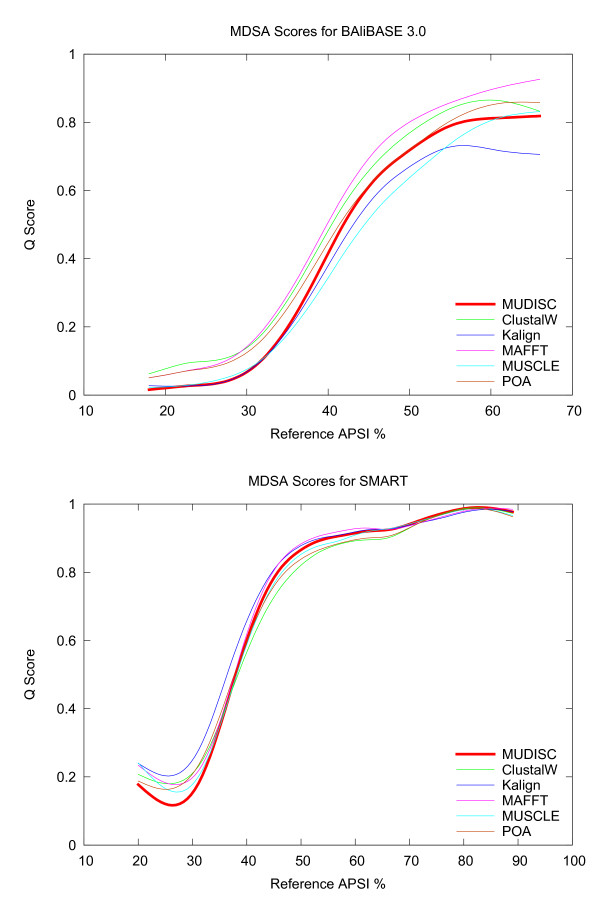
**Alignment quality on the MDSA data set**. MUDISC (the new method) is compared with several alignment programs on the MDSA data set which contains nucleotide adaptations of the BAliBASE and SMART reference alignments. BAliBASE includes reference sets 1-7. MUDISC uses discrete profile alignment.

A comparison of alignment quality with the MDSA reference sets is reported in Figure 11. The Q score, which is equivalent to the sum-of-pairs score (SPS) score [38], is shown in relation to the APSI. Unlike the FI, the Q score only considers residue pairs correctly aligned in the test alignment compared with the reference and does not count residue-gap pairs. The acsplines smoothing option is again used, but with a weighting factor of 1e-2. MUDISC is on par with other MSA programs down to about 40% APSI and is still comparable in accuracy below 40% APSI.

The average alignment quality of MUSCLE and MUDISC is shown in Table 2. A variant of MUDISC that uses the nearest neighbor search method is also shown. According to the Friedman rank test [39] with an adjustment for ties, the difference in quality between the near and nearest neighbor search methods is not significant. Even though the average scores are very similar, the difference between MUSCLE and MUDISC is significant with MUSCLE ranking higher on BRAliBase and MUDISC ranking higher on the MDSA data set.

**Table 2:** Comparison of MUSCLE and MUDISC alignment quality

Reference Set	MUSCLE Avg. Score	P-value, Rank	MUDISC (near) Avg. Score	P-value, Rank	MUDISC (nearest) Avg. Score
BRAliBase k7	0.6846	1.42e-4, >	0.6851	0.875, >	0.6839
BRAliBase k15	0.6914	1.18e-3, >	0.6819	0.285, <	0.6814
MDSA BAliBASE	0.3125	9.58e-9, <	0.3623	0.806, <	0.3629
MDSA SMART	0.6195	7.46e-3, <	0.6295	0.830, >	0.6316

The average quality scores for MUSCLE and MUDISC are shown on four reference sets. BRAliScore is reported for BRAliBase, and Q score is reported for MDSA. A variant of MUDISC that uses the nearest neighbor search method is also shown. The P-values from a Friedman rank test indicate the difference between two adjacent programs, and the relational symbols indicate which program ranked higher.

Program run times for MUSCLE, MUDISC, and MUFPGA are reported in Table 3. MUFPGA obtains an overall speedup of 33 relative to MUSCLE on the Influenza data set and a speedup of 154 on the HIV data set. Run times for MUFPGA on the Corona and Herpes data sets are estimated since the accelerator currently only supports sequence lengths up to 16 K. This maximum length is a function of the FPGA register sizes and the amount of memory configured to buffer intermediate sequences and is not limited by the method. The estimated run times for MUFPGA show the benefits of discrete profile alignment on long sequences when pairwise alignment is significantly accelerated. To calculate these values, the pairwise alignment time in MUDISC is reduced by a factor of 290, which is extrapolated from timings on the Influenza and HIV data sets. Pairwise alignment in the third stage is accelerated by a factor of 176 on the Influenza data set and a factor of 283 on the HIV data set. Our prior work [4] has characterized the pairwise alignment performance of the FPGA accelerator and has shown a speedup over 300 relative to a desktop computer.

**Table 3:** Alignment program run times and overall speedup

Data Set	Sequences	Avg. Length	MUSCLE	MUDISC	MUFPGA	Speedup
Influenza	12,104	1,717	01:25:40	00:20:03	00:02:35	33
HIV	2,144	9,019	02:09:29	01:28:42	00:00:50	154
Corona	400	29,531	03:17:47	02:22:43	00:00:41*	284*
Herpes	142	167,043	136:38:20	52:31:05	00:11:26*	716*

* Estimated

Program run times are in HH:MM:SS.

**Figure 12:**
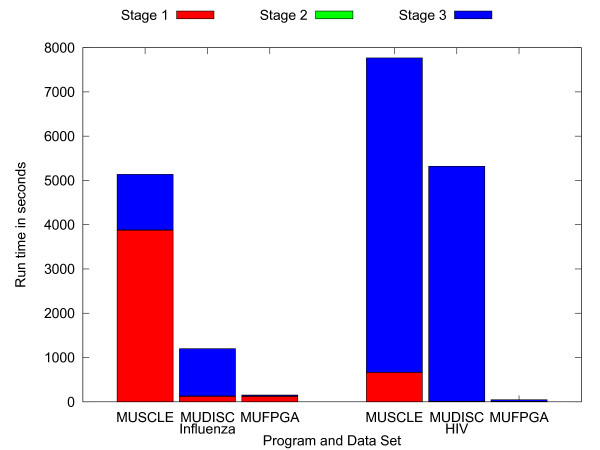
**Alignment run time comparison with stages**. Overall program runtimes are shown on the Influenza and HIV data sets with a breakdown of time spent in each stage.

Figure 12 shows the proportion of time spent in the three stages of alignment on the Influenza and HIV data sets. The time for each stage includes both iterations. SSE acceleration improves the first stage run time with a speedup of 31 on the Influenza data set and a speedup of 79 on the Corona data set. Notice that the proportion of time spent in similarity calculations on the Influenza data set is greater with more sequences and limits the overall speedup. The Stage 3 run time of MUFPGA is faster than MUSCLE by a factor of 60 on the Influenza data set and by a factor of 198 on the HIV data set. The profile reduction rate ranges from 44.1 to 98.1 Mpos/s and the reduction time ranges from 5.4 to 11.7% of the pairwise alignment time on the accelerator.

## Conclusions

The discrete profile alignment algorithm presented here produces alignments with quality comparable to other leading MSA programs and enables the acceleration of progressive alignment. A speedup over 150 is demonstrated when discrete profile alignment is combined with an FPGA accelerator that uses a fine-grained parallel approach for the DP calculations of pairwise alignment. Previous coarse-grained approaches are limited by insufficient parallelism, particularly in the third stage of MSA. The discrete profile alignment algorithm in conjunction with a fast pairwise alignment algorithm advance the capabilities and performance of large-scale MSA. A key component of our method is a fast profile reduction algorithm on the host that can supply sequences at a rate comparable to the accelerator's input data rate. The reduction algorithm uses a near neighbor search in hyper-dimensional profile space to quantize profile positions at a rate up to 100 Mpos/s on a single core. Since this rate is sufficient to support the high-end performance of reconfigurable computing, other acceleration methods based on GPUs or SSE instructions may also be a viable option.

Minimizing the time for sequence similarity calculations in Stage 1 is also important to achieve significant speedup, especially for data sets with large numbers of sequences. Using SSE instructions reduces the time for sequence similarity calculations by a factor of 30-80. Thousands of sequences can be aligned in a few minutes when Stage 1 is accelerated with SSE instructions and Stage 3 is accelerated with reconfigurable computing.

Future work includes the extension of discrete profile alignment to support proteins that have a larger alphabet by adding more dimensions to profile space. Since proteins have an amino acid alphabet of 20 characters, instead of 4 like DNA, an alphabet compression scheme would be necessary to reduce the number of characters and the corresponding dimensionality of profile space to a practical number. Reducing the alphabet to six classes based on physico-chemical properties, as done in MAFFT, would only require 6 dimensions for the classes plus 1 for gaps.

Another area of investigation is to apply the coarse-grained parallelism of cluster supercomputers and the fine-grained parallelism of reconfigurable computing to multiple sequence alignment. Since this work only uses a single core and one accelerator, a cluster with reconfigurable computing could provide an estimated 20-30x speedup beyond this work.

## Authors' contributions

SL conceived of the study, carried out the experiments, performed the comparative analysis and drafted the manuscript. QS supervised the research, participated in the design of the study and assisted in the manuscript preparation. All authors read and approved the final manuscript.
